# Antimicrobial Activity of Human Prion Protein Is Mediated by Its N-Terminal Region

**DOI:** 10.1371/journal.pone.0007358

**Published:** 2009-10-07

**Authors:** Mukesh Pasupuleti, Markus Roupe, Victoria Rydengård, Krystyna Surewicz, Witold K. Surewicz, Anna Chalupka, Martin Malmsten, Ole E. Sörensen, Artur Schmidtchen

**Affiliations:** 1 Division of Dermatology and Venereology, Department of Clinical Sciences, Lund University, Lund, Sweden; 2 Division of Infection Medicine, Department of Clinical Sciences, Lund University, Lund, Sweden; 3 Department of Physiology and Biophysics, Case Western Reserve University, Cleveland, Ohio, United States of America; 4 Department of Pharmacy, Uppsala University, Uppsala, Sweden; Johns Hopkins School of Medicine, United States of America

## Abstract

**Background:**

Cellular prion-related protein (PrP^c^) is a cell-surface protein that is ubiquitously expressed in the human body. The multifunctionality of PrP^c^, and presence of an exposed cationic and heparin-binding N-terminus, a feature characterizing many antimicrobial peptides, made us hypotesize that PrP^c^ could exert antimicrobial activity.

**Methodology and Principal Findings:**

Intact recombinant PrP exerted antibacterial and antifungal effects at normal and low pH. Studies employing recombinant PrP and N- and C-terminally truncated variants, as well as overlapping peptide 20mers, demonstrated that the antimicrobial activity is mediated by the unstructured N-terminal part of the protein. Synthetic peptides of the N-terminus of PrP killed the Gram-negative bacteria *Escherichia coli* and *Pseudomonas aeruginosa*, and the Gram-positive *Bacillus subtilis* and *Staphylococcus aureus*, as well as the fungus *Candida parapsilosis*. Fluorescence studies of peptide-treated bacteria, paired with analysis of peptide effects on liposomes, showed that the peptides exerted membrane-breaking effects similar to those seen after treatment with the “classical” human antimicrobial peptide LL-37. In contrast to LL-37, however, no marked helix induction was detected for the PrP-derived peptides in presence of negatively charged (bacteria-mimicking) liposomes. PrP furthermore showed an inducible expression during wounding of human skin *ex vivo* and *in vivo*, as well as stimulation of keratinocytes with TGF-α in vitro.

**Conclusions:**

The demonstration of an antimicrobial activity of PrP, localisation of its activity to the N-terminal and heparin-binding region, combined with results showing an increased expression of PrP during wounding, indicate that PrPs could have a previously undisclosed role in host defense.

## Introduction

The innate immune system, based on antimicrobial peptides (AMP), provides a rapid and non-specific response against potentially invasive pathogenic microorganisms. At present, over 900 different AMP peptide sequences are known[1], [3] (see also http://www.bbcm.univ.trieste.it/~tossi/amsdb.html). The majority of AMPs are characterized by an amphipathic structure, composed of hydrophobic and cationic amino acids spatially organized in sectors of the molecules. For example, AMPs comprise linear peptides, many of which may adopt α-helical and amphipathic conformation upon bacterial binding, peptides forming cysteine-linked antiparallel β-sheets, as well as cysteine-constrained loop structures. AMPs may also, however, be found among peptides not displaying such ordered structures as long as these are characterized by an over-representation of certain amino acids[1], [4], [5], [6]. The interaction with bacterial membranes is a prerequisite for AMP function. However, the modes of action of AMPs on their target bacteria are complex, and can be divided into membrane disruptive and non-membrane disruptive[1], [3], [7], [8].

It has become increasingly clear, that AMPs belong to a multifunctional group of molecules that interact with negatively charged glycosaminoglycans (such as heparin), biomembranes, and cell receptors. Apart from their antibacterial actions, biological effects exerted by AMPs include growth stimulus and angiogenesis, protease inhibition, anti-angiogenesis, and chemotaxis[9], [10], [11]. Conversely, cationic peptide motifs from proteins not previously considered as AMPs have been shown to exert antimicrobial activities. For example, the anaphylatoxin peptide C3a and kininogen-derived peptides exert antimicrobial effects[12], [13], [14], [15]. In conjunction with these findings, consensus heparin-binding peptide sequences were shown to be antibacterial[16] and specifically interact with membranes[17].

Cellular prion-related protein (PrP^c^) is a cell-surface protein that is ubiquitously expressed in the human body. Apart from a high expression in the brain, the protein is also found throughout the body in blood, skin[18], haematopoietic cells[19], gastric mucosa [20], mammary glands and kidney[21]. Although numerous biological functions have been attributed to prions, their exact physiological role is still not known. Hence, recent work has suggested that PrP^c^ is required for self-renewal of hematopoietic cells[19] and may be involved in T-cell activation[21]. PrP^c^ is highly expressed in the neural system, and since this is the major site of prion pathology, most interest has been focused on defining the role of PrP^c^ in neurons. However, PrPc−/− mice are relatively normal, only presenting subtle abnormalities in synaptic transmission[21]. Accumulated evidence suggests that PrPc may function as a metal transporter, and binding of Cu^2+^ also enables PrPc to acquire a superoxide-dismutase like antioxidant activity[22], [23]. Other ligands that have been reported to bind PrPc include laminin, nucleic acids, as well as glycosaminoglycans such as heparin[24]. Considering the latter it was demonstrated that the heparin-binding site was located in the N-terminus of PrP^c^, involving amino acid residues 23–35. Interestingly, an increase in prion protein expression has been demonstrated during bacterial infection[20] and inflammation[18], and hence it has been hypothesized that PrP^c^ may act as a pattern recognition receptor inducing the innate and adaptive immunity in a TLR independent manner[25].

The multi-functionality of PrP^c^, and presence of an exposed cationic and heparin-binding N-terminus made us raise the question whether PrP^c^ could exert antimicrobial activity. We here show that the recombinant prion protein may indeed kill both Gram-negative and Gram-positive bacteria and fungi, the activity being mediated by the N-terminus of PrP. These findings, together with a marked induction of PrP expression in skin wounds, suggest that antimicrobial activity could be one potential role of the prion protein.

## Materials and Methods

### Peptides

Pep-screen peptides were from Sigma-Genosys, generated by a peptide synthesis platform (PEPscreen, Custom Peptide Libraries, Sigma Genosys) and yield was ∼1–6 mg with an average crude purity of 60–70%. Prior to biological testing, the peptides were diluted in dH2O (5 mM stock) and stored at −20°C. This stock solution was used for the subsequent experiments. The high quality peptides LVL20; LVLFVATWSDLGLCKKRPKP, KKR20; KKRPKPGGWNTGGSRYPGQG, MAN28; MANLGCWMLVLFVATWSDLGLCKKRPKP, GHH20; GHHPHGHHPHGHHPHGHHPH and AHH24; AHHAHAAHH AHAAHHAHAAHHAHA were synthesized by Biopeptide Co., San Diego, USA, with the exception of LL-37, which was obtained from Innovagen AB, Lund, Sweden. The purity (>95%) of these peptides was confirmed by mass spectral analysis (MALDI-ToF Voyager). The production of recombinant PrP and truncated versions has been described previously [26]. Human TGF-α was from Peprotech (Rocky Hill, NJ). The polyclonal goat antibody against prion protein (PrP_27–30_), used for imunnohistochemistry and immunoblotting, was purchased from Chemicon.

### Microorganisms

Bacterial isolates *Escherichia coli* ATCC 25922, *Pseudomonas aeruginosa* ATCC 27853, *Staphylococcus aureus* ATCC 29213, *Bacillus subtilis* ATCC 6633, *Candida albicans* ATCC 90028 and *Candida parapsilosis* ATCC 90018 and were obtained from the Department of Bacteriology, Lund University Hospital.

### Viable count analysis

*Escherichia coli* ATCC 25922 was grown overnight in full-strength (3% w/v) trypticase soy broth (TSB) (Becton-Dickinson, Cockeysville, MD), whereas *C. parapsilosis* ATCC 90018 was grown in YPD medium. The microbes were washed twice with 10 mM Tris, pH 7.4, and diluted in 10 mM Tris, pH 7.4, 5 mM glucose or in 10 mM MES pH 5.5, containing 5 mM glucose. Following this, microbes (in 50 µl; 1−2×10^6^ cfu/ml) were incubated at 37°C for 2 hours at the indicated concentrations with PrP and variants thereof, or with peptides in appropriate buffers in presence/absence of 0.15 M NaCl or 20% citrate plasma. In some experiments, *C. parapsilosis* were incubated with peptides or recPrp in 10 mM Tris, pH 7.4 mM glucose in the absence or presence of 50 µM Zn^2+^. For analyses in buffers mimicking human sweat (40 mM NaCl, 10 mM KCl, 1 mM CaCl_2_, 1 mM MgCl_2_ and 1 mM Na-dihydrogenphosphate, pH 5.5 or 6.5), *C. parapsilosis* (1−2×10^7^ cfu/ml) was incubated with recPrP (1 µM) in sweat buffer for 2 hours at 37°C as described previously[27]. To quantify the bactericidal activity, serial dilutions of the incubated mixtures were plated on TH (for bacteria), or YPD agar (for fungi), followed by incubation at 37°C overnight and the number of colony-forming units was determined. 100% survival was defined as total survival of bacteria in the same buffer and under the same conditions as in the absence of peptide. Significance was determined using the statistical software SigmaStat (SPSS Inc., Chicago, IL, USA).

### Radial diffusion assay

Essentially as described earlier [28], bacteria were grown overnight in 10 ml of full-strength (3% w/v) trypticase soy broth (TSB) (Becton-Dickinson, Cockeysville, MD), whereas fungi were grown in YPD medium and washed twice with 10 mM Tris, pH 7.4. 4×10^6^ colony forming units was added to 15 ml of the underlay agarose gel, consisting of 0.03% (w/v) TSB, 1% (w/v) low-electro endosmosis type (Low-EEO) agarose (Sigma, St Louise MO) and a final concentration of 0.02% (v/v) Tween 20 (Sigma). Three different underlay gels were used, each based on different compositions (10 mM Tris, pH 7.4, 10 mM MES, pH 5.5, and 10 mM Tris, pH 7.4, 50 µM Zn^2+^). The underlay was poured into a Ø 144 mm petri dish. After agarose solidification, 4 mm-diameter wells were punched and 6 µl of peptide was added to each well. Plates were incubated at 37°C for 3 hours to allow diffusion of the peptides. The underlay gel was then covered with 15 ml of molten overlay (6% TSB and 1% Low-EEO agarose in dH_2_O). Antimicrobial activity of a peptide was visualized as a clear zone around each well after 18–24 hours of incubation at 37°C.

### Fluorescence microscopy

The impermeant probe FITC (Sigma-Aldrich, St. Louis, USA) was used for monitoring of bacterial membrane permeabilization. *S. aureus* ATCC 29213 were grown to mid-logarithmic phase in TSB medium. Bacteria were washed and resuspended in buffer (10 mM Tris, pH 7.4, 0.15 M NaCl, 5 mM glucose) to yield a suspension of 1×10^7^ CFU/ml. 100 µl of the bacterial suspension was incubated with 30 µM of the respective peptides at 30°C for 30 min. Microorganisms were then immobilized on poly (L-lysine)-coated glass slides by incubation for 45 min at 30°C, followed by addition onto the slides of 200 µl of FITC (6 µg/ml) in buffer and a final incubation for 30 min at 30°C. The slides were washed and bacteria fixed by incubation, first on ice for 15 min, then in room temperature for 45 min in 4% paraformaldehyde. The glass slides were subsequently mounted on slides using Prolong Gold antifade reagent mounting medium (Invitrogen, Eugene, USA). Bacteria were visualized using a Nikon Eclipse TE300 (Nikon, Melville, USA) inverted fluorescence microscope equipped with a Hamamatsu C4742-95 cooled CCD camera (Hamamatsu, Bridgewater, USA) and a Plan Apochromat ×100 objective (Olympus, Orangeburg, USA). Differential interference contrast (Nomarski) imaging was used for visualization of the microbes themselves.

### Hemolysis assay

EDTA-blood was centrifuged at 800 g for 10 min, whereafter plasma and buffy coat were removed. The erythrocytes were washed three times and resuspended in PBS, pH 7.4 to get a 5% suspension. The cells were then incubated with end-over-end rotation for 60 min at 37°C in the presence of peptides (60 µM). 2% Triton X-100 (Sigma-Aldrich) served as positive control. The samples were then centrifuged at 800 g for 10 min and the supernatant was transferred to a 96 well microtiter plate. The absorbance of hemoglobin release was measured at λ 540 nm and is in the plot expressed as % of TritonX-100 induced hemolysis.

### Lactate dehydrogenase (LDH) assay

HaCaT keratinocytes were grown to confluency in 96 well plates (3000 cells/well) in serum-free keratinocyte medium (SFM) supplemented with bovine pituitary extract and recombinant EGF (BPE-rEGF) (Invitrogen, Eugene, USA). The medium was then removed, and 100 µl of the peptides investigated (at 60 µM, diluted in SFM/BPE-rEGF or in keratinocyte-SFM supplemented with 20% human serum) were added. The LDH-based TOX-7 kit (Sigma-Aldrich, St. Louis, USA) was used for quantification of LDH release from the cells. Results represent mean values from triplicate measurements, and are given as fractional LDH release compared to the positive control consisting of 1% Triton X-100 (yielding 100% LDH release).

### Slot-blot assay

LPS binding ability of the peptides were examined by slot-blot assay. Peptides (2 and 5 µg) were bound to nitrocellulose membrane (Hybond-C, GE Healthcare BioSciences, UK), pre-soaked in PBS, by vacuum. Membranes were then blocked by 2 wt% BSA in PBS, pH 7.4, for 1 h at RT and subsequently incubated with ^125^I-labelled LPS (40 µg/mL; 0.13×10^6^ cpm/µg) or ^125^I-labelled heparin (Sigma) for 1 h at RT in 10 mM Tris, pH 7.4, 0.15 M NaCl, or 10 mM MES, pH 5.5, 0.15 M NaCl. After LPS binding, membranes were washed 3 times, 10 min each time in the above buffers and visualized for radioactivity on Bas 2000 radioimaging system (Fuji, Japan).

### Liposome preparation and leakage assay

The liposomes investigated were either zwitterionic (DOPC/cholesterol 60/40 mol/mol or DOPC without cholesterol) or anionic (DOPE/DOPG 75/25 mol/mol). DOPG (1,2-Dioleoyl-*sn*-Glycero-3-Phosphoglycerol, monosodium salt), DOPC (1,2-dioleoyl-*sn*-Glycero-3-phoshocholine), and DOPE (1,2-dioleoyl-*sn*-Glycero-3-phoshoetanolamine) were all from Avanti Polar Lipids (Alabaster, USA) and of >99% purity, while cholesterol (>99% purity), was from Sigma-Aldrich (St. Louis, USA). Due to the long, symmetric and unsaturated acyl chains of these phospholipids, several methodological advantages are reached. In particular, membrane cohesion is good, which facilitates very stable, unilamellar, and largely defect-free liposomes (observed from cryo-TEM) and well defined supported lipid bilayers (observed by ellipsometry and AFM), allowing detailed values on leakage and adsorption density to be obtained. The lipid mixtures were dissolved in chloroform, after which solvent was removed by evaporation under vacuum overnight. Subsequently, 10 mM Tris buffer, pH 7.4, was added together with 0.1 M carboxyfluorescein (CF) (Sigma, St. Louis, USA). After hydration, the lipid mixture was subjected to eight freeze-thaw cycles consisting of freezing in liquid nitrogen and heating to 60°C. Unilamellar liposomes of about Ø140 nm were generated by multiple extrusions through polycarbonate filters (pore size 100 nm) mounted in a LipoFast miniextruder (Avestin, Ottawa, Canada) at 22°C. Untrapped CF was removed by two subsequent gel filtrations (Sephadex G-50, GE Healthcare, Uppsala, Sweden) at 22°C, with Tris buffer as eluent. CF release from the liposomes was determined by monitoring the emitted fluorescence at 520 nm from a liposome dispersion (10 mM lipid in 10 mM Tris, pH 7.4). An absolute leakage scale was obtained by disrupting the liposomes at the end of each experiment through addition of 0.8 mM Triton X-100 (Sigma-Aldrich, St. Louis, USA). A SPEX-fluorolog 1650 0.22-m double spectrometer (SPEX Industries, Edison, USA) was used for the liposome leakage assay, and also for monitoring W absorption spectra of GKH17-WWW in Tris buffer in the absence and presence of liposomes under conditions described above. Measurements were performed in triplicate at 37°C.

### CD-spectroscopy

The CD spectra of the peptides in solution were measured on a Jasco J-810 Spectropolarimeter (Jasco, U.K.). The measurements were performed at 37°C in a 10 mm quartz cuvet under stirring and the peptide concentration was 10 µM. The effect on peptide secondary structure of liposomes at a lipid concentration of 100 µM was monitored in the range 200–250 nm. The only peptide conformations observed under the conditions investigated were α-helix and random coil. The fraction of the peptide in α -helical conformation, X_α_, was calculated from
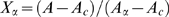
where A is the recorded CD signal at 225 nm, and A_α_ and A_c_ are the CD signal at 225 nm for a reference peptide in 100% α-helix and 100% random coil conformation, respectively. 100% α -helix and 100% random coil references were obtained from 0.133 mM (monomer concentration) poly-L-lysine in 0.1 M NaOH and 0.1 M HCl, respectively[29], [30]. For determination of effects of lipopolysaccharide on peptide structure, the peptide secondary structure was monitored at a peptide concentration of 10 µM, both in Tris buffer and in the presence of *E. coli* lipopolysaccharide (0.02 wt%) (*Escherichia coli* 0111:B4, highly purified, less than 1% protein/RNA, Sigma, UK). To account for instrumental differences between measurements the background value (detected at 250 nm, where no peptide signal is present) was subtracted. Signals from the bulk solution were also corrected for.

### Keratinocyte cultures

Primary human keratinocytes were obtained from Cascade Biologics (Portland, OR) and cultured in serum-free medium (KGM2-Bullet kit) from Cambrex (Walkersville, MD). 24 hours after complete confluence was reached cells were stimulated with TGF-α (50 ng/mL) for 48 hours or left non-stimulated for control before harvesting.

### Human skin wounds

Non-wounded human skin was obtained by taking punch biopsies from three donors, while skin wound samples were retrieved by making new punch biopsies from the edges of the initial biopsies. A small fraction of these samples were fixed in formalin for immunohistochemistry or prepared for miccroarray analysis as previously described[31]. The material was obtained under protocols approved by the Ethics Committee at Lund University, Lund, Sweden.

### Model of *ex vivo* injured human skin

Surplus, normal, skin was obtained as previously described from three donors following surgery according to protocols approved by the Ethics Committee at Lund University. In brief the skin was cut into slices of 1×10 mm and incubated in culture for 4 days (*ex vivo* injured skin). The skin samples were cultured in serum-free keratinocyte medium (KGM2-Bullet kit) from Cambrex (Walkersville, MD) supplemented with transferrin, hEGF (0.15 ng/mL), 0.5 mg/mL hydrocortisone, gentamicin, amphotericin B, and epinephrine but without insulin (all supplied by Cambrex).

### RNA isolation and microarray analysis

Total RNA was isolated with Trizol (Invitrogen, Carlsbad, CA) according to the recommendations of the supplier and resuspended in 0.1 mmol/L EDTA. The concentration was determined by spectrophotometric measurement. For gene expression analysis, total RNA was biotinylated and hybridized to Human Genome U133 Plus 2.0 GeneChips® (Affymetrix, Santa Clara, CA) according to the instructions by the manufacturer. The microarray fluorescence signals were normalized using the GeneChip Operation Software (GCOS ver. 1.4, Affymetrix). All probe set lists were annotated with locus link identifications (IDs) provided by the NetAffx database and converged into gene/EST (expressed sequence tag) lists by exclusion of redundant probe sets with identical locus ID. Genes were defined as expressed in cell populations if all replicates were assigned a present call by the GCOS software (Affymetrix). Genes of potential interest for wound healing were therefore only genes with at least three present calls in either the *in vivo* control condition or in the *in vivo* wound condition. The microarray data present in this study has been sent in the MAIME (Minimum Information About a Microarray Experiment) compliant MAGE-TAB format to the ArrayExpress database (www.ebi.ac.uk/arrayexpress) where it soon will be made publicly available under an accession number[32].

### Immunohistochemistry

The wound specimens (described earlier) that were fixed in 10% formalin, were dehydrated and embedded in paraffin. Sections of 5 µm thickness were placed on poly-lysine coated glass slides, deparaffinized in xylene and rehydrated in graded alcohols. The slides were then treated with Dako antigen retrieval solution (Dako) for 40 min at 97°C. The slides were incubated for 24 hours at room temperature in a 1∶1000 dilution of polyclonal antibodies (adcam, England) The antibodies were diluted in TBS with 1% BSA, 5% serum from the same species as the secondary antibody, 0.05% Tween 20 (Sigma). After three 20 min washes in TBS with 0.05% Tween 20 the slides were incubated with alkaline phosphatase conjugated secondary anti IgG (Dako) diluted 1∶1000 in the same buffer as the first antibody and incubated for another 24 hours followed by three 20 min washes. Color was developed with Vulcan Fast Red chromogen (Biocare Medical, Concord, CA) and the slides were counterstained with Harris Hematoxylin (EM Science, Gibbstown, NJ).

### SDS-PAGE and immunoblotting

SDS-PAGE and immunoblotting were performed according to the instructions from the manufacturer (BioRad, Hercules, CA). After transfer of proteins from the polyacrylamide gels, the PVDF-membrane was fixed for 30 min in TBS with 0.05% glutaraldehyde (Sigma) and blocked with 3% skimmed milk. For visualization of the poly(peptides), polyvinylidene difluoride (PVDF) membranes were incubated overnight with primary antibodies. The following day, the membranes were incubated for 2 hours with HRP-conjugated secondary antibodies (Dako, Glostrup, Denmark) and visualized by SuperSignal West Pico Chemiluminescent Substrate (Thermo Scientific, Rockford, IL). The PVDF membrane was stripped for 20 minutes in 0.2 mmol/L glycine (pH 2.5), 1% SDS, washed twice with TBS with 0.05% Tween-20 and finally blocked with 3% skimmed milk before incubating overnight with a different antibody.

### Statistics

Values are reported as means±standard deviation of the means. To determine significance, analysis of variance with ANOVA (SigmaStat, SPSS Inc., Chicago, USA), followed by *post hoc* testing using the Holm-Sidak method, or Student's t-test, were used as indicated in the figure legends, where “n” denotes number of independent experiments. Significance was accepted at p<0.05.

### Ethics Statement

This study was conducted according to the principles expressed in the Declaration of Helsinki. The study was approved by the Institutional Review Board of Lund University hospital. Written informed consent for the collection of samples and subsequent analysis was obtained.

## Results

**Figure 1:**
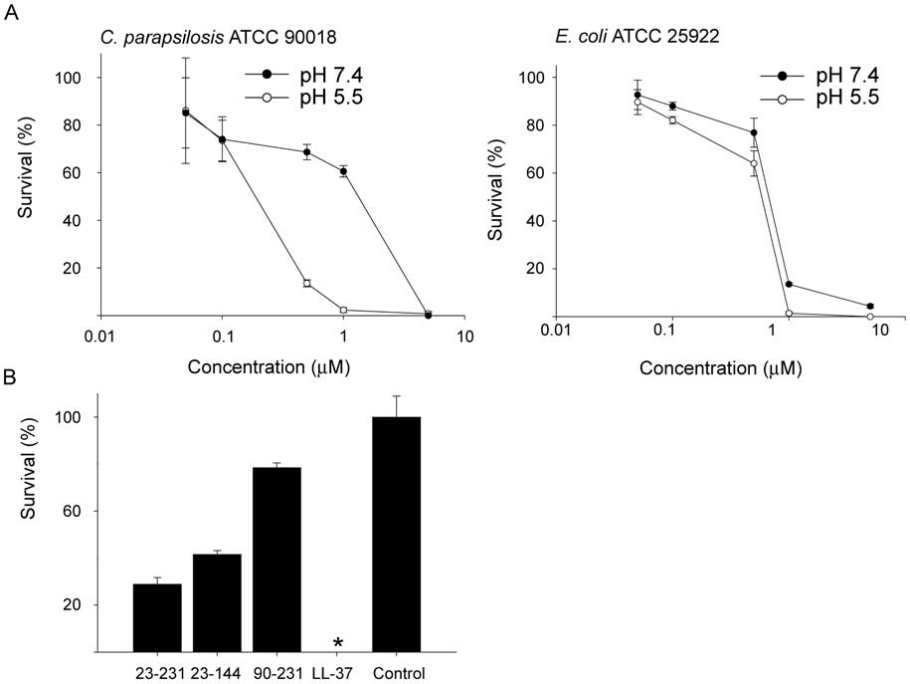
Antimicrobial effects of recPrP and variants. (A) In viable count assays, *Escherichia coli* and *Candida parapsilosis* were subjected to increasing doses of PrP in 10 mM Tris pH 7.4 (left panel) or 10 mM MES pH 5.5 (both containing 5 mM glucose) and the number of cfu was determined. The values represent mean of triplicate samples, and a representative experiment (of three) is shown. The difference in activity against *C. parapsilosis* at 1 uM is statistically significant (pooling of three experiments yielded P<0.001, n = 9, t-test) (B) Comparison of antibacterial effects of PrP with truncated variants. *E. coli* was incubated with 1 µM of the full-length PrP protein, or with variants PrP_23–144_ or PrP_90–231_. LL-37 is shown for comparison.

**Figure 2:**
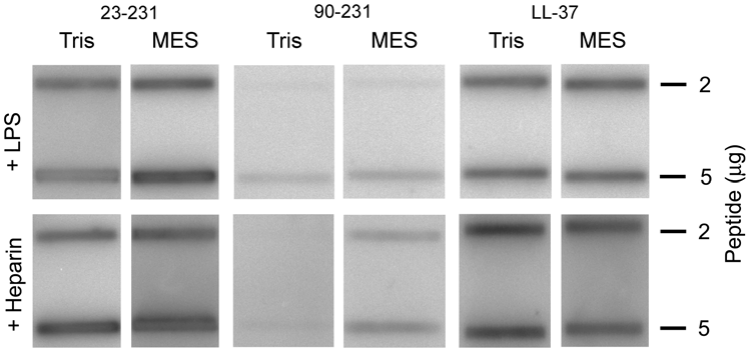
Interactions of PrP with LPS and heparin. 2 and 5 µg of recPrP and the truncated PrP_90–231_ were applied onto nitrocellulose membranes, followed by incubation with iodinated (^125^I) heparin or LPS in either Tris pH 7.4 or MES pH 5.5 (all at 10 mM, with 0.15 M NaCl). Radioactivity of bound heparin or LPS was visualized using a phosphorimager system. LL-37 is included for comparison.

To investigate whether human prion protein could exert antimicrobial effects, we tested the activity of protein against the Gram-negative *E. coli* and the fungus *C. parapsiliosis*. The protein was shown to be antimicrobial against these microbes at normal (pH 7.4) as well as low pH (pH 5.5). An increase in antimicrobial activity, particularly against *C. parapsilosis*, was noted at low pH (Fig. 1A, for positive controls, see Figure S1). In order to explore which part of PrP that could harbour the antimicrobial activity, the full-length PrP protein (containing amino acids 23–231) was compared with two truncated forms, either lacking the C-terminal part (PrP_23–144_), or with a N-terminal truncated form (PrP_90–231_)(Fig. 1B). Since the latter form, lacking the first 90 amino acids of PrP significantly lost its antimicrobial potential, the results indicated that the major antimicrobial activity was dependant on an intact N-terminal region of PrP. In analogy with the antimicrobial activity, slot-binding experiments with iodinated LPS showed that intact PrP bound LPS at normal as well as low pH, contrasting to the result with the N-terminally truncated form (Fig. 2). Notably, PrP bound LPS similarly to human LL-37. Experiments using iodinated heparin paralleled the findings with LPS, and confirmed that the N-terminal region of PrP mediates heparin binding (Fig. 2). Furthermore, heparin completely blocked the interaction with LPS as well as antimicrobial activity, confirming that heparin interacting sequences of PrP mediate LPS binding, and thus, antimicrobial activity (not shown).

**Figure 3:**
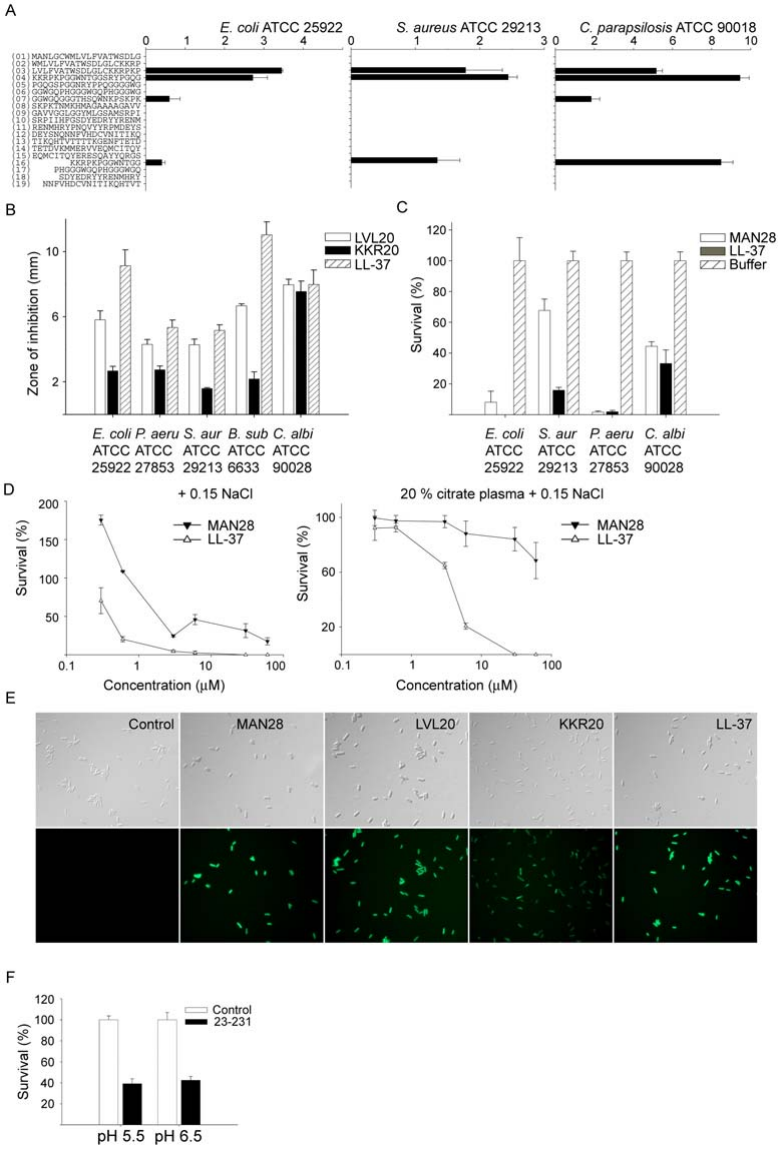
Activities of peptide sequences of PrP. (A) Antimicrobial activity of selected peptides (at 100 µΜ in RDA) against the indicated microbes. For determination of antimicrobial activities, *E. coli* ATCC 25922, *S. aureus* ATCC 29213 isolates (4×10^6^ cfu) or *C. parapsilosis* ATCC 90018 (1×10^5^ cfu) was inoculated in 0.1% TSB agarose gel. Each 4 mm-diameter well was loaded with 6 µl of peptide. The zones of clearance correspond to the inhibitory effect of each peptide after incubation at 37 °C for 18–24 h (mean values are presented, n = 3). (B) Antimicrobial activity (at 100 µΜ in RDA) against a panel of microbes of the selected highly pure peptides LVL20 and KKR20, and comparison with LL-37. (C) In viable count assays, the indicated microbes were subjected to the N-terminal PrP peptide MAN28 (at 30 µΜ) in 10 mM Tris pH 7.4 containing 5 mM glucose. Identical buffers without peptide were used as controls. (D) In viable count assays, *E. coli* bacteria were subjected to the indicated peptides in 10 mM Tris pH 7.4 containing 0.15 M NaCl in absence or presence of 20% human citrate-plasma. Identical buffers without peptide were used as controls. (E) Permeabilizing effects of peptides on *E. coli*. *E. coli* was incubated with the indicated peptides and permeabilization was assessed using the impermeant probe FITC. (F) Effects of recPrP (23–231) in presence of buffers mimicking the salt content and pH of human sweat. In viable count assays, *Candida parapsilosis* were subjected to PrP (1 µΜ) in sweat buffer at pH 5.5 or 6.5 and the number of cfu was determined.

In order to further explore the structure-function relationship of epitopes of PrP, overlapping peptide sequences comprising 20mers, as well as shorter variants (Fig. 3A, see also Figure S2 for illustration of sequence and peptides) of PrP were synthesized and screened against the Gram-negative *E. coli*, Gram-positive *S. aureus* and the fungus *Candida parapsilosis* using radial diffusion assays under low salt conditions. The experiments demonstrated that the antimicrobial activity was mainly found in two regions comprising the N-terminal peptide containing the previously identified heparin-binding motif of PrP[24]; KKRPK (peptides no: 3 and 4, and 16 in the list in Fig. 3A). It has been reported that the signal sequence of PrP^c^, normally cleaved of by a signal peptidase, may be retained, leading to the secretion of unprocessed protein forms[33]. Notably, the KKRPK motif, containing a part of the signal sequence (peptide no: 3), was also antimicrobial. We have previously shown that antimicrobial activities of histidine-rich regions, such as those found in kininogen and histidine-rich glycoprotein, rely on the presence of Zn^2+^ or low pH [34], [35]. Considering that stretches of PrP are histidine-rich, and given the slight increase of PrP-mediated antimicrobial activity against *C. parapsilosis* at low pH (Fig. 1A), we next investigated the activity of the 20mers in RDA at pH 5.5. Whereas the previously described H-rich peptides GHHPHGHHPHGHHPHGHHPH (GHH20), derived from histidine-rich glycoprotein [36] and AHHAHAAHHAHAAHHAHAAHHAHA (AHH24)[37] both showed enhanced activity at low pH, the PrP-derived 20mers yielded no additional clearance zones at low pH (Figure S3A, B). In viable count assays, Zn^2+^ at 50 µM did not enhance the activity of PrP (Figure S4A), nor were additional antimicrobial 20mer peptides detected using RDA in the presence of Zn^2+^ (Figure S4B,C).

Although valuable for the initial evaluation, the PEP peptides were only used for initial screening purposes and for selection of putative active peptides. Still, inspection of the mass-spectrometry data showed that contaminants consisted of smaller peptides, being truncated version of the major peptide of relatively minor effect on the observed antimicrobial activity[38]. Based on the data obtained from the initial screening, highly pure (>95%) peptides were synthesized and further analysed; the previously described LVLFVATWSDLGLCKKRPKP (LVL20), KKRPKPGGWNTGGSRYPGQG (Fig. 3A), but also a longer form extending from the N-terminus and encompassing the KKRPKP sequence; MANLGCWMLVLFVATWSDLGLCKKRPKP (MAN28). Antimicrobial assays confirmed the high activity of these peptides, when compared to the benchmark peptide LL-37 (Figure 3B, C). First, the activity of the peptides LVL20 and KKR20 was analysed using RDA. These peptides all exerted antimicrobial activities against the above microbes, as well as *P. aeruginosa* and *B. subtilis* (Fig. 3B). It was noted that the activities were comparable to those demonstrated for the classical antimicrobial cathelicidin peptide LL-37. Previous results have shown that the peptides derived from the unprocessed N-termini of mouse and bovine prion proteins, comprising hydrophobic sequences followed by the charged domain KKRPKP, cause membrane perturbation and lysis of phospholipid vesicles[39] When tested in RDA, MAN28 yielded no antimicrobial activity (not shown). However, highly hydrophobic peptides may aggregate/and or associate[40], or interact with the matrix, in both cases leading to underestimation of the antimicrobial activity[38]. Hence, the peptide was tested in viable count assays. The results showed that the peptide indeed had a capacity to kill both bacteria and fungi (Fig. 3C). It is well known that activities of AMPs are dependent of the microenvironment. For example, various chemokines, defensins, as well as LL-37 are partly, or completely, antagonized by high salt conditions or the presence of plasma proteins *in vitro* [41], [42]. The results showed that MAN28 retained some antimicrobial activity in 0.15 M NaCl, however, its activity was lost in the presence of plasma proteins (Fig. 3D). The former LVL20 and KKR20 were inactive in the presence of salt and plasma. The membrane permeabilizing effects of MAN28, LVL20, and KKR20 are illustrated in Figure 3E, where an increased uptake of the impermeant dye propidium iodide is noted after subjection of *E. coli* to the peptides. Similarly to the above peptides, PrP was also inactive in the presence of salt and plasma (not shown). It should be noted, however, that the protein retained some activity in buffers reflecting the salt and pH environment of human skin[27] (Fig. 3F).

**Figure 4:**
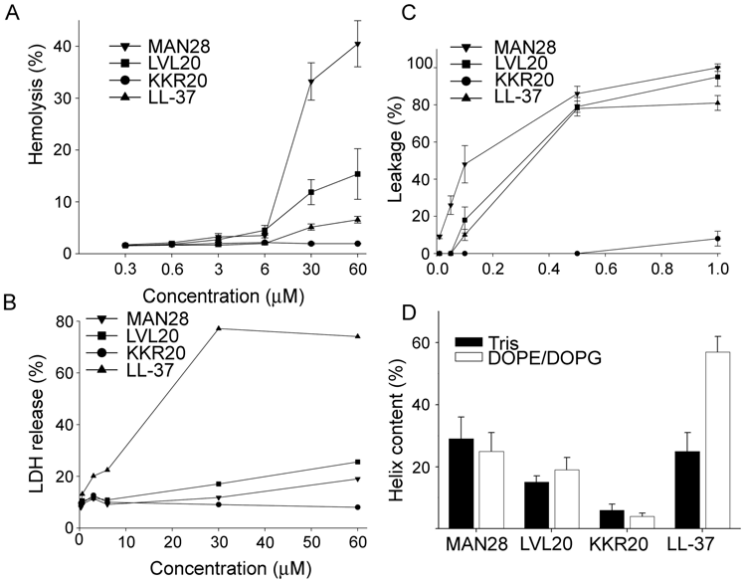
Activities on eukaryotic cells and liposomes. (A) Hemolytic effects of MAN28, LVL20, and KKR20 were investigated. The cells were incubated with different concentrations of the peptides, 2% Triton X-100 (Sigma-Aldrich) served as positive control. The absorbance of hemoglobin release was measured at λ 540 nm and is expressed as % of Triton X-100 induced hemolysis (note the scale of the y-axis). Effects of LL-37 are shown for comparison. (B) HaCaT keratinocytes were subjected to the indicated peptides prion-derived peptides as well as LL-37. Cell permeabilizing effects were measured by the LDH based TOX-7 kit. LDH release from the cells was monitored at λ 490 nm and was plotted as % of total LDH release. (C) Effects of the indicated peptides on liposome leakage. The membrane permeabilizing effect was recorded by measuring fluorescence resulting from the release of carboxyfluorescein from negatively charged DOPE/DOPG liposomes. Values represents mean of triplicate samples. (D) Helical content of the indicated peptides in the presence or absence of negatively charged liposomes (DOPE/DOPG). Only LL-37 showed a marked helix induction upon addition of the liposomes.

AMPs that kill bacteria may also exhibit hemolytic and membrane permeabilising activities against eukaryotic cells. It was noted that particularly MAN28, but also to lesser extent LVL20, both containing the hydrophobic signal sequence, displayed hemolytic effects which exceeded those observed for LL-37 (Fig. 4A). Furthermore, the peptide MAN28 significantly permeabilized HaCat cells (a keratinocyte cell line), whereas LL-37 as well as LVL20 where less active in this respect (Fig. 4B). In liposome models, the peptides containing the KKRPKP sequence caused CF release thus indicating a direct effect on lipid membranes (Fig. 4C). In contrast to the classical helical peptides LL-37, circular dichroism showed that there was no helix induction upon incubation of the peptides with (negatively charged) DOPG/DOPG liposomes (Fig. 4D).

**Figure 5:**
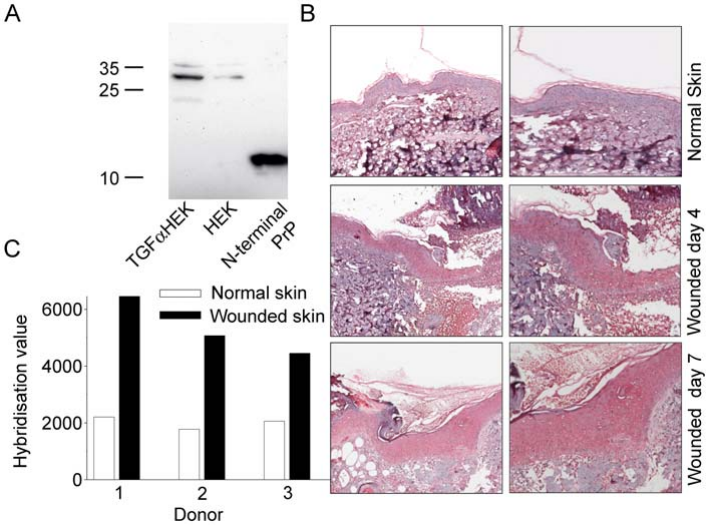
Expression and microarray hybridization levels of PrP in wounded skin *ex vivo* and *in vivo*. (A) PrP expression in keratinocyte cultures. Cell extracts from keratinocytes grown in absence or presence of TGF-α were subjected to SDS-PAGE, blotted and probed with antibodies to PrP 27–30. recPrP_23–144_ (0.5 µg) was used as positive control. (B) Expression of PrP in wounds *in vivo*. Normal skin biopsies and biopsies of wound edges (at day 4) were immunostained for PrPc. Hematoxylin was used for counterstaining. (C) Hybridization levels of PRPN in *ex vivo* wounded and non-wounded skin after four days in culture are presented.

Next, western blot was performed on extracts of keratinocyte cultures stimulated with TGF-α. A stronger signal for PrP was noted demonstrating that increased amounts of PrP are produced by hyperproliferative keratinocytes (Fig. 5A). Immunohistochemistry with antibodies against the N-terminal part of PrP showed a prominent staining of PrP that was particularly apparent 4 days after wounding as compared to the low amounts found in normal non-wounded epidermis (Fig 5B). Furthermore, hybridization values for PrP were investigated in a microarray experiment performed on *in vivo* skin wounds. In wounded skin of all three donors, high levels of Prp expression were detected, when compared to unwounded skin (not shown). Additional microarray experiment using skin wounded *ex vivo* (n = 3) yielded similar results (p-value = 0.005) (Figure 5C). These data clearly demonstrate an inducible expression of PrP during inflammatory/hyperproliferative conditions in human skin *ex vivo* and *in vivo*.

## Discussion

The key findings in this study are the identification of an antimicrobial activity of PrP together with the characterization of epitopes mediating this effect, as well as mechanistic data showing that the antimicrobial activity is mediated by bacterial membrane permeabilisation. This new property of PrP is in line with several observations indicating that heparin-binding proteins, attributed various roles in biology, such as complement C3[13], kininogen[12], [15], heparin-binding protein[43], heparin-binding epidermal growth factor and other growth factors[44], β2-glycoprotein[45], histidine-rich glycoprotein[36], may, either as holoproteins or after fragmentation, also exert antimicrobial activities i*n vitro*, and in several cases, *in vivo* [12], [15], [36] .

Mature PrP^c^ contains a folded globular C-terminal domain and an N-terminal region which is largely unstructured[46]. From a structural perspective, several lines of evidence suggest that the antimicrobial activity relies on the sequence KKRPK in the unstructured N-terminal domain. However, since this domain also contains stretches of histidines, as well as other sequence motifs, mediating interactions with Zn^2+^ and Cu^2+^, it cannot be completely excluded that additional antimicrobial effects of PrP may be mediated by this region, particularly at low pH and perhaps in concert with contributions from additional helical motifs in the globular part. Having said this, investigations addressing this, e.g. testing effects of the overlapping 20mer peptides at low pH as well as in presence of Zn^2+^, did not detect any additional antimicrobial regions. However, it should be noted that the 20mers obviously show limitations with respect to both length and structure, and may not reflect the possible contribution of the H-rich region to the observed antimicrobial activity of intact PrP. With respect to the N-terminal signal peptides containing the KKRPKP sequence, MAN28 (and its shorter variant LVL20) it is particularly interesting that related peptides of mouse and bovine origin permeabilize liposomes, in agreement with the antibacterial activity reported here on the human peptide sequence. The findings that the N-terminal peptide of mature PrP (KKR20), as well as recombinantly produced PrP is antimicrobial, indicate however, that the signal peptide is not required for antimicrobial activity of the protein. Considering the N-terminal peptide KKR20, the peptide likely resembles other linear peptides having a low helical content. For example, AMPs derived from growth factors display a low helical content in buffer and in presence of membranes, reflecting their low content of features typical of “classical” helical peptides, such as regularly interspersed hydrophobic residues[44]. Furthermore, studies utilizing ellipsometry, CD, fluorescence spectroscopy, and z-potential measurements on a kininogen-derived antimicrobial peptide, HKH20 (HKHGHGHGKHKNKGKKNGKH)[47] showed that the HKH20 peptide display primarily random coil conformation in buffer and at lipid bilayers, the interactions dominated by electrostatics, as evidenced by strongly reduced adsorption and membrane rupture at high ionic strength[47] Both HKH20[12] and GKR22 (GKRKKKGKGLGKKRDPCLRKYK) [44], a peptide derived from heparin-binding growth factor, retain antibacterial activity in physiological buffers as well as in plasma. In contrast to these observations, the prion protein-derived KKR20 lost its antibacterial activity at high salt concentrations (0.15 M NaCl), illustrating that cationicity alone is not a sufficient parameter for determining antimicrobial activity of a given peptide, and exemplifying that amphipathicity, as well as hydrophobicity, enabling bacterial membrane interactions, are necessary for activity of many AMPs[1], especially at physiological salt concentrations, where initial electrostatic interactions with anionic cell wall components are diminished by the ionic environment.

A number of mechanisms by which AMPs induce membrane defects have been observed. For some peptides, e.g., melittin, alamethicin, magainin 2 and gramicidin A [7], [48], [49], [50], as well as the N-terminal PrP-derived signal peptides of mouse and bovine origin [39], transmembrane structures have been reported, forming transient pores. For disordered and highly charged peptides membrane disruption is obtained by other mechanisms, e.g., induction of a negative curvature strain, membrane thinning, or local packing defects associated with peptide localization primarily in the phospholipid polar headgroup region[7], [17], [47], [51], [52], [53]. Although the data reported here demonstrate that peptide-induced bacterial membrane damage correlates to membrane defect formation in a model lipid membrane system, additional studies are warranted in order to study the exact mode of action of human PrP and related peptides.

As previously mentioned, many antimicrobial peptides, including LL-37[54], C3a [13], and kininogen derived peptides [12], [15] are released during proteolysis. Whether N-terminal fragments of the prion protein are generated *in vivo* remains to be investigated. However, the observation that cleavage of the N-terminal part of PrP occur in response to oxidative stress and reactive oxygen species (ROS), releasing low molecular weight fragments of about 6 kDa [55], point at the interesting possibility that antimicrobial fragments of the prion proteins may be released during inflammation. Furthermore, although evidence suggest that extracellular PrP may be released from cells [56], research is needed whether this shed PrP may reach sufficiently high antimicrobial levels *in vivo*. It should also be noted, that PrP, as well as the studied peptides (with the exception of the signal peptide, MAN28) lost their antimicrobial activity at high salt strength, casting doubt of the potential antimicrobial role of PrP *in vivo*. Nevertheless, there is now convincing evidence that AMPs, such as similarly salt-sensitive defensins and chemokines[57], [58] contribute to enhanced bacterial killing *in vivo*, likely reflecting the necessity of their compartmentalization, presence of ionic microenvironments (as illustrated by retained activity of PrP in sweat buffers), or synergism between AMPs.

As mentioned above, prion protein is not only confined to the nervous system, but instead ubiquitously found in many other cells and tissues, and the physiological role for this protein are still enigmatic. In a previous study, it was reported that human keratinocytes express PrP^c^ *in vitro* and during inflammatory skin disease [18]. Although that previous work was focusing on prion infectivity routes, our current findings on increased expression of PrP during wounding, together with the observation of its antimicrobial activity, clearly indicate that PrPs could have a previously undisclosed role in host defense. In this context, experiments with PrP deficient animals in infection models should be valuable in order to further delineate a possible role of PrP in innate defense.

## Supporting Information

Figure S1 Antimicrobial effects of histidine-rich peptides at low pH. In viable count assays, Candida parapsilosis were subjected to increasing doses of the peptides AHH24; AHHAHAAHH AHAAHHAHAAHHAHA (left panel) and GHH20; GHHPHGHHPHGHHPHGHHPH (right panel) in 10 mM Tris pH 7.4 or in 10 mM MES pH 5.5 and the number of cfu was determined.
(0.40 MB TIF)

Figure S2 Sequence of PrP and overlapping 20 mer peptides. The peptides used in the study are indicated. In addition to the overlapping peptides, regions of specific interest, eg. high charge, and content of helical structures were selected.
(0.35 MB TIF)

Figure S3 Activities of peptide sequences of PrP at normal and low pH. (A) Antimicrobial activity of selected peptides (at 100 uM in RDA) against C. parapsilosis ATCC 90018 (1×105 cfu). The fungi were inoculated in a 0.1% TSB agarose gel containing 10 mM Tris, pH 7.4 or 10 mM MES, pH 5.5. Each 4 mm-diameter well was loaded with 6 ul of peptide. The zones of clearance correspond to the inhibitory effect of each peptide after incubation at 37°C for 18–24 h (mean values are presented, n = 3). (B) In a similar setup as above, the control peptides AHH24; AHHAHAAHHAHAAHHAHAAHHAHA and GHH20; GHHPHGHHPHGHHPHGHHPH were tested at the indicated doses. The activity of these control peptides was enhanced at low pH.
(0.97 MB TIF)

Figure S4 Activities of PrP and derived peptide sequences in absence and presence of Zn2+. (A) In viable count assays, Candida parapsilosis was subjected to PrP at 1 mM in 10 mM Tris pH 7.4 in absence and presence of 50 uM Zn2+, and the number of cfu was determined (n = 3). There was no significant difference in PrP activity in absence and presence of Zn2+. The peptides AHH24; AHHAHAAHH AHAAHHAHAAHHAHA (center panel) and GHH20; GHHPHGHHPHGHHPHGHHPH (right panel) showed no antimicrobial activity in 10 mM Tris, but showed a dose-dependent killing of C. parapsilosis in presence of 50 uM Zn2+. (B) Antimicrobial activity of PrP-derived peptides (at 200 uM in RDA) against C. parapsilosis ATCC 90018 (1×105 cfu). The fungi were inoculated in a 0.1% TSB agarose gel containing 10 mM Tris, pH 7.4 with or without 50 uM Zn2+. Each 4 mm-diameter well was loaded with 6 ul of peptide. The zones of clearance correspond to the inhibitory effect of each peptide after incubation at 37°C for 18–24 h (mean values are presented, n = 3). (C) In a similar setup as in B, the control peptides AHH24 and GHH20 were tested at 200 uM in RDA. The activity of these control peptides was significantly enhanced at low pH (n = 3, p<0.05).
(1.25 MB TIF)
