# Supplementary figures and images for: Antimicrobial Activity of Human Prion Protein Is Mediated by Its N-Terminal Region
Source: PLoS One. 2009 Oct 7;4(10):e7358. doi: 10.1371/journal.pone.0007358 (PMC2752989; doi:10.1371/journal.pone.0007358)

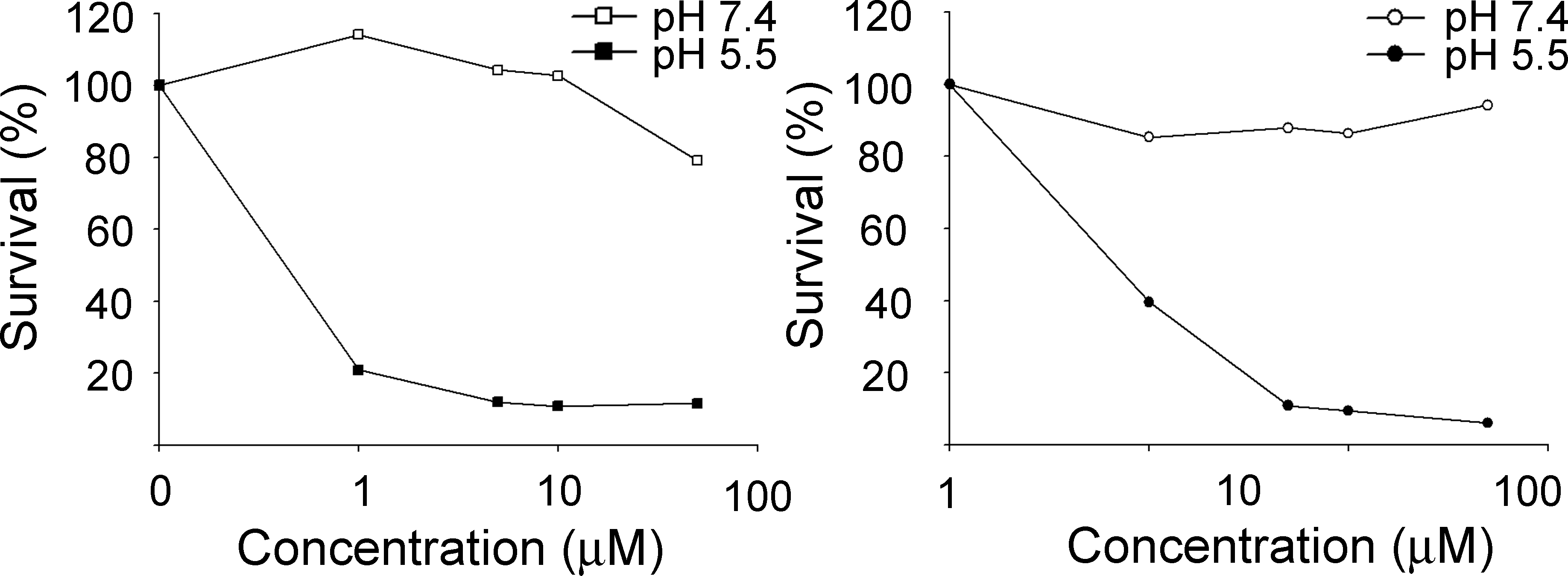

Supplement: Figure S1 — Antimicrobial effects of histidine-rich peptides at low pH. In viable count assays, Candida parapsilosis were subjected to increasing doses of the peptides AHH24; AHHAHAAHH AHAAHHAHAAHHAHA (left panel) and GHH20; GHHPHGHHPHGHHPHGHHPH (right panel) in 10 mM Tris pH 7.4 or in 10 mM MES pH 5.5 and the number of cfu was determined. (0.40 MB TIF) [file pone.0007358.s001.tif]

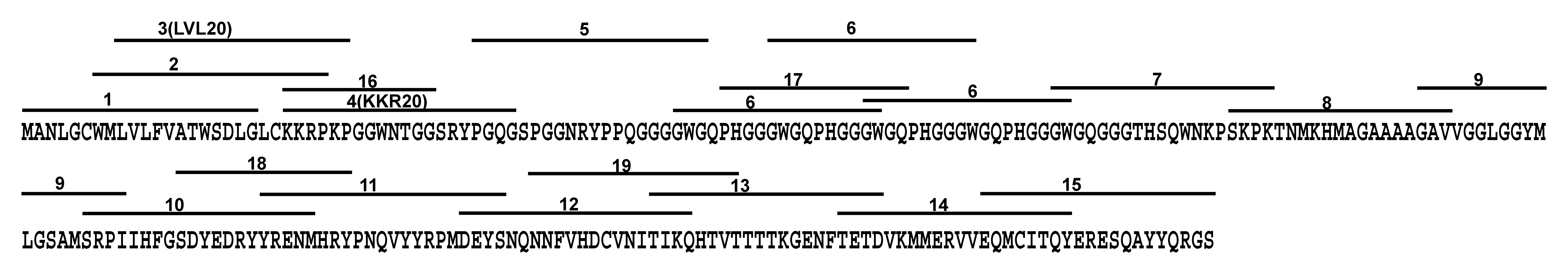

Supplement: Figure S2 — Sequence of PrP and overlapping 20 mer peptides. The peptides used in the study are indicated. In addition to the overlapping peptides, regions of specific interest, eg. high charge, and content of helical structures were selected. (0.35 MB TIF) [file pone.0007358.s002.tif]

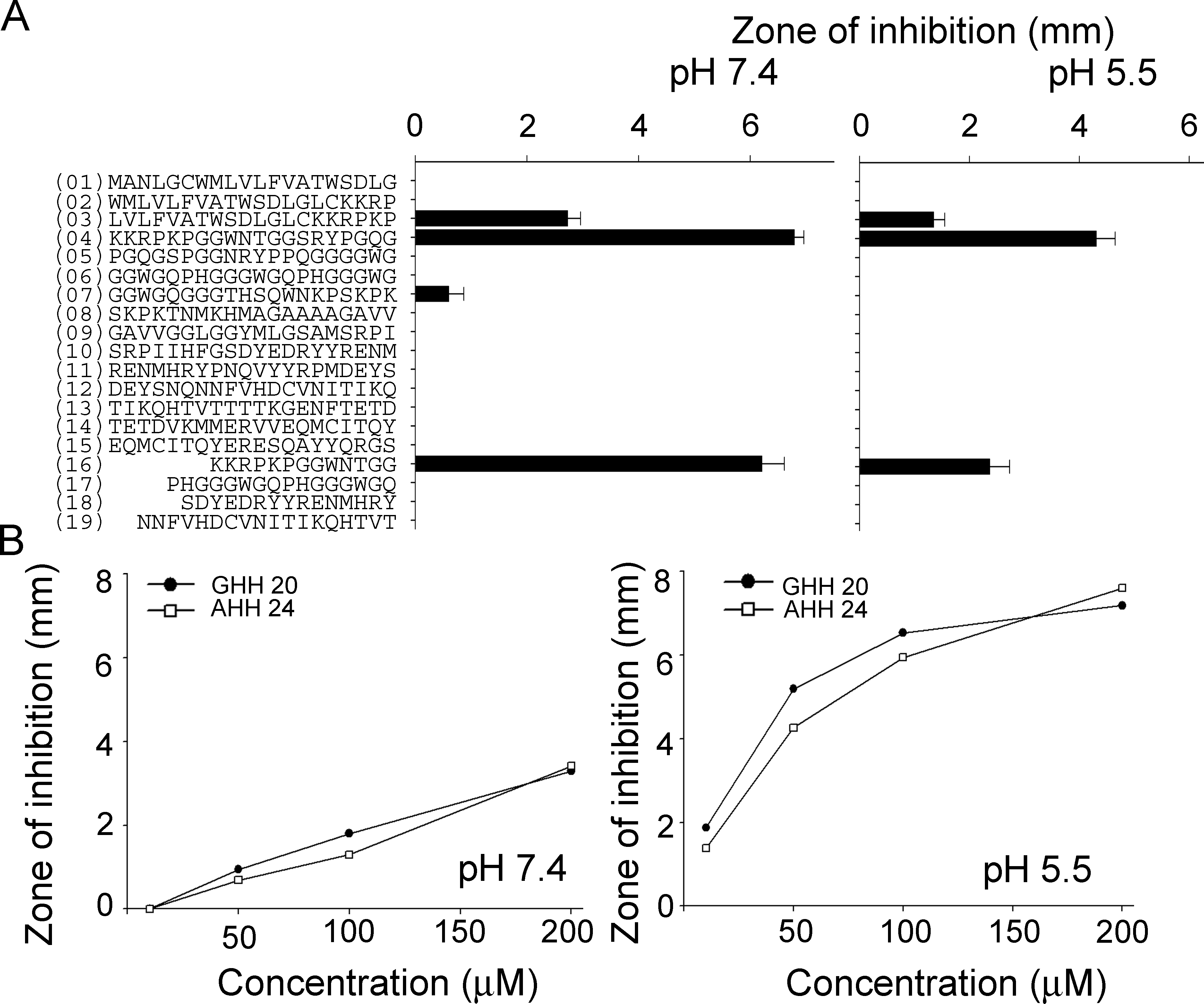

Supplement: Figure S3 — Activities of peptide sequences of PrP at normal and low pH. (A) Antimicrobial activity of selected peptides (at 100 uM in RDA) against C. parapsilosis ATCC 90018 (1×10^5^ cfu). The fungi were inoculated in a 0.1% TSB agarose gel containing 10 mM Tris, pH 7.4 or 10 mM MES, pH 5.5. Each 4 mm-diameter well was loaded with 6 ul of peptide. The zones of clearance correspond to the inhibitory effect of each peptide after incubation at 37°C for 18–24 h (mean values are presented, n = 3). (B) In a similar setup as above, the control peptides AHH24; AHHAHAAHHAHAAHHAHAAHHAHA and GHH20; GHHPHGHHPHGHHPHGHHPH were tested at the indicated doses. The activity of these control peptides was enhanced at low pH. (0.97 MB TIF) [file pone.0007358.s003.tif]

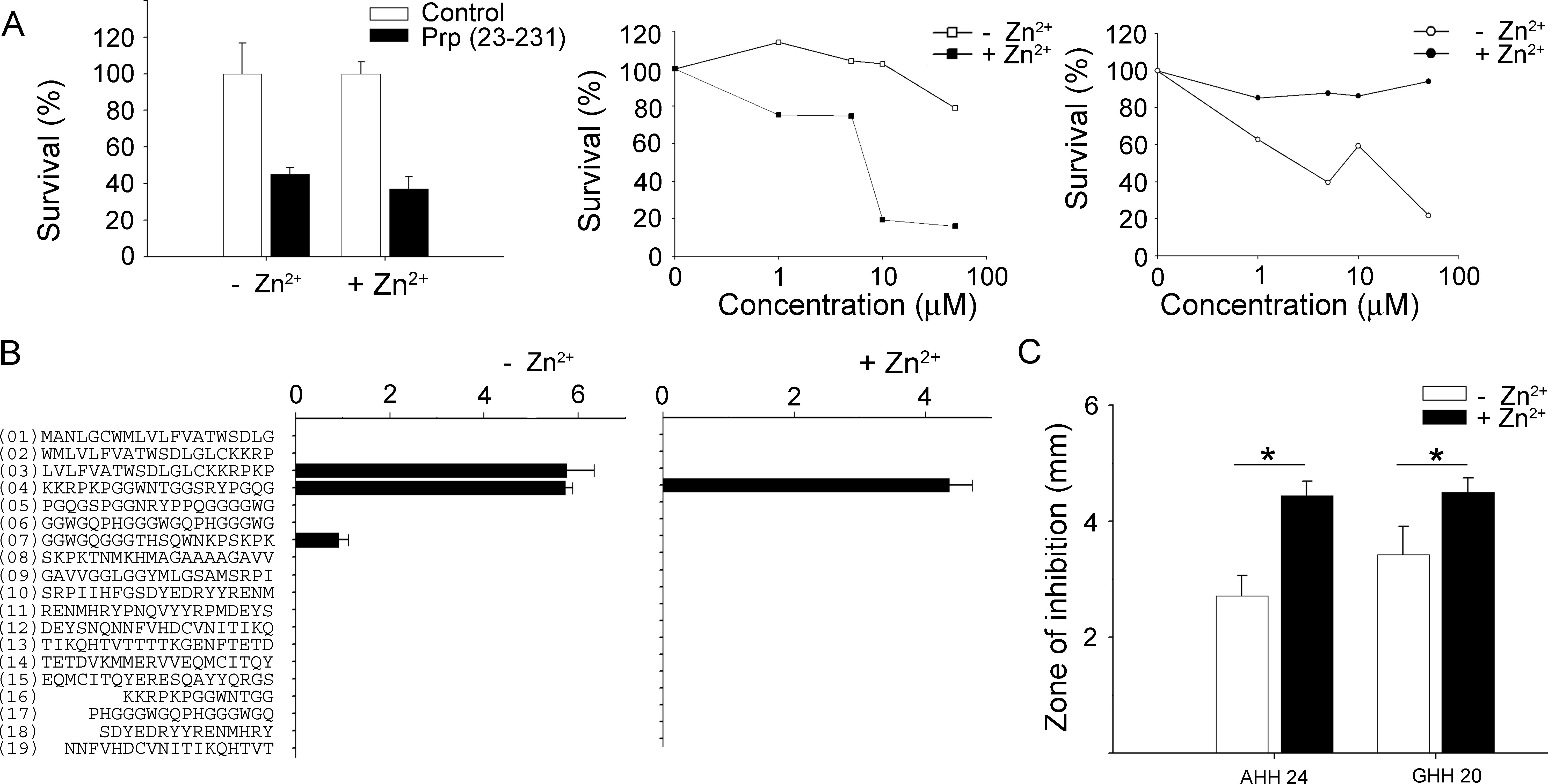

Supplement: Figure S4 — Activities of PrP and derived peptide sequences in absence and presence of Zn2+. (A) In viable count assays, Candida parapsilosis was subjected to PrP at 1 mM in 10 mM Tris pH 7.4 in absence and presence of 50 uM Zn2+, and the number of cfu was determined (n = 3). There was no significant difference in PrP activity in absence and presence of Zn2+. The peptides AHH24; AHHAHAAHH AHAAHHAHAAHHAHA (center panel) and GHH20; GHHPHGHHPHGHHPHGHHPH (right panel) showed no antimicrobial activity in 10 mM Tris, but showed a dose-dependent killing of C. parapsilosis in presence of 50 uM Zn2+. (B) Antimicrobial activity of PrP-derived peptides (at 200 uM in RDA) against C. parapsilosis ATCC 90018 (1×10^5^ cfu). The fungi were inoculated in a 0.1% TSB agarose gel containing 10 mM Tris, pH 7.4 with or without 50 uM Zn2+. Each 4 mm-diameter well was loaded with 6 ul of peptide. The zones of clearance correspond to the inhibitory effect of each peptide after incubation at 37°C for 18–24 h (mean values are presented, n = 3). (C) In a similar setup as in B, the control peptides AHH24 and GHH20 were tested at 200 uM in RDA. The activity of these control peptides was significantly enhanced at low pH (n = 3, p<0.05). (1.25 MB TIF) [file pone.0007358.s004.tif]
